# A Novel Approach to Assess Weekly Self-efficacy for Meeting Personalized Physical Activity Goals Via a Cellphone: 12-Week Longitudinal Study

**DOI:** 10.2196/38877

**Published:** 2023-01-27

**Authors:** Yoo Jung Oh, Thomas J Hoffmann, Yoshimi Fukuoka

**Affiliations:** 1 Department of Communication University of California, Davis Davis, CA United States; 2 Department of Epidemiology and Biostatistics University of California, San Francisco San Francisco, CA United States; 3 Department of Physiological Nursing University of California, San Francisco San Francisco, CA United States

**Keywords:** self-efficacy, physical activity, exercise, cellphone, mobile phone, application, app, Ecological Momentary Assessment

## Abstract

**Background:**

Despite the health benefits of engaging in regular physical activity (PA), the majority of American adults do not meet the PA guidelines for aerobic and muscle-strengthening activities. Self-efficacy, the belief that one can execute specific actions, has been suggested to be a strong determinant of PA behaviors. With the increasing availability of digital technologies, collecting longitudinal real-time self-efficacy and PA data has become feasible. However, evidence in longitudinal real-time assessment of self-efficacy in relation to objectively measured PA is scarce.

**Objective:**

This study aimed to examine a novel approach to measure individuals' real-time weekly self-efficacy in response to their personalized PA goals and performance over the 12-week intervention period in community-dwelling women who were not meeting PA guidelines.

**Methods:**

In this secondary data analysis, 140 women who received a 12-week PA intervention were asked to report their real-time weekly self-efficacy via a study mobile app. PA (daily step counts) was measured by an accelerometer every day for 12 weeks. Participants rated their self-efficacy on meeting PA goals (ranging from “not confident” to “very confident”) at the end of each week via a mobile app. We used a logistic mixed model to examine the association between weekly self-efficacy and weekly step goal success, controlling for age, BMI, self-reported White race, having a college education or higher, being married, and being employed.

**Results:**

The mean age was 52.7 (SD 11.5, range 25-68) years. Descriptive analyses showed the dynamics of real-time weekly self-efficacy on meeting PA goals and weekly step goal success. The majority (74.4%) of participants reported being confident in the first week, whereas less than half of them (46.4%) reported confidence in the final week of the intervention. Participants who met weekly step goals were 4.41 times more likely to be confident about achieving the following week's step goals than those who did not meet weekly step goals (adjusted odds ratio 4.41; 95% CI 2.59-7.50; *P*<.001). Additional analysis revealed that participants who were confident about meeting the following week’s step goals were 2.07 times more likely to meet their weekly step goals in the following week (adjusted odds ratio 2.07; 95% CI 1.16-3.70; *P*=.01). The significant bidirectional association between real-time self-efficacy and weekly step goal success was confirmed in a series of sensitivity analyses.

**Conclusions:**

This study demonstrates the potential utility of a novel approach to examine self-efficacy in real time for analysis of self-efficacy in conjunction with objectively measured PA. Discovering the dynamic patterns and changes in weekly self-efficacy on meeting PA goals may aid in designing a personalized PA intervention. Evaluation of this novel approach in an RCT is warranted.

## Introduction

Regular physical activity (PA) reduces the risk of chronic diseases such as heart disease, hypertension, type 2 diabetes, and depression [1]; improves quality of life [2]; and prevents weight gain [3]. Despite these known health benefits, approximately 80% of American adults currently do not meet the PA guidelines for aerobic and muscle-strengthening activities [4]. Women, in particular, are less likely to be physically active than men in the United States [5,6]. Thus, it is critical to understand the determinants of engagement in regular PA and develop effective interventions to encourage PA at the population level.

Self-efficacy, a promising indicator for PA interventions, is one’s belief in one’s capacity to execute specific actions [7]. Individuals with high self-efficacy are considered more likely to engage in regular PA by overcoming potential obstacles and setbacks. Traditionally, changes to self-efficacy following PA interventions are measured only at a single or limited set of time points (eg, baseline and at 12 weeks). This traditional model of intermittent assessment is cost-effective and feasible, but does not capture the dynamic nature of changes to self-efficacy over time and could delay intervening against relapse [8].

The increasing availability of digital technologies (eg, smartphone applications and activity trackers) has facilitated longitudinal collection of real-time self-efficacy and PA data [9,10]. For example, Ecological Momentary Assessment (EMA) allows researchers to gather repeated real-time participant reports of momentary cognitive and behavioral states in naturalistic settings [8]. EMA, compared with conventional cross-sectional data collection methods, has several advantages, including minimized recall bias and the ability to collect timestamped longitudinal data. In research on addictions to tobacco, gambling, or alcohol, EMA has been employed to capture fluctuations in the participants' cravings and self-efficacy and to intervene against relapse in real time [11,12]. However, unlike in addiction-related research, use of EMA for real-time assessment of self-efficacy for PA is somewhat limited. In particular, real-time data for self-efficacy in relation to PA, along with continuous collection of objectively measured PA data, are scarce.

Thus, this study aimed to examine a novel approach to measure individuals' real-time weekly self-efficacy in response to their personalized PA goals and performance. Using a mobile app that both delivers a PA intervention and collects data on self-efficacy, we sought to discover an association between self-efficacy and PA among female participants who were previously physically inactive. To the best of our knowledge, this is the first study to examine real-time self-efficacy using EMA in relation to objectively measured PA every day for the 12-week intervention period.

## Methods

### Study Design

This study was conducted via secondary analysis of the data collected from the PA intervention group during the first 12 weeks of a mobile phone–based PA educational trial, an unblinded, parallel, randomized controlled trial (RCT). Detailed protocol, sample size calculation, and the main results of this RCT are published elsewhere [13-16]. This RCT was based on the social cognitive theory that emphasizes dynamic interactions between personal factors and behaviors and their environments [17]. The main outcome study results have been previously published [18].

### Ethical Considerations

The trial was approved by the University of California, San Francisco Committee on Human Research (#10-04566) and the Data and Safety Monitoring Board. All participants signed the informed consent and the Health Insurance Portability and Accountability Act form before participating in this study. Participants received up to US $80, a study tote bag, and a study T-shirt upon completing all study requirements.

### Study Participants

In brief, the eligibility criteria were female sex, age between 25 and 69 years, Body Mass Index (BMI) of 18.5-43.0 kg/m^2^, being physically inactive (ie, did not meet PA recommendations) at work and during leisure time based on the Stanford Brief Activity Survey [19], intending to be physically active, having access to a home telephone or mobile phone, being able to speak and read English, absence of medical conditions or physical complications requiring special attention in an exercise program, and no current participation in other lifestyle modification programs. Written informed consent was obtained from all participants.

### Intervention

During the 12-week intervention period, the participants in the intervention group received a PA intervention, which consisted of brief in-person counseling sessions at randomization, 6 weeks, and 12 weeks, as well as weekly administration of the study trial app created by the research team. A detailed description of both components has been published previously [14]. The in-person counseling sessions encompassed seven domains: (1) overview of the PA program and tailored short- and long-term goal setting based on each participant's baseline PA data; (2) education about the duration and intensity of brisk walking and the health benefits of PA; (3) identification of barriers to increasing PA and development of strategies to overcome these barriers; (4) value and identification of social support while increasing PA; (5) relapse prevention; (6) education about healthy diet and weight maintenance; and (7) PA safety. A written individualized PA plan was developed during the initial in-person counseling session immediately after randomization and then re-evaluated at the 6-week and 12-week visits. The trial app consisted of two main functions: (1) a daily message or video clip and (2) a daily diary. The daily messages or video clips reinforced the 7 domains addressed in the brief in-person intervention. A preprogrammed daily message or video clip was sent once a day at a predetermined time between 11 AM and 3 PM. The daily PA diary was accessible between 7 PM and midnight. If no diary entry was made by 8:30 PM, an automated text message was sent to remind the participant to record their total daily steps and the type and duration of PA performed. An automated message was also sent if a participant did not use the app for 3 consecutive days. Activity goals displayed under the “weekly goals” option were automatically increased by 20% each week, relative to the participant's run-in average, until a goal of 10,000 steps per day, 7 days a week, was reached. For example, if the participant had an average of 5000 steps per day during the run-in period, the first, second, and third week of the daily step goal would be 6000 steps, 7200 steps, and 8640 steps, respectively. In week 4 and afterward, the daily step goal would stay at 10,000 steps. After the intervention started, no step goal adjustment was made regardless of participants’ performance. In this study, we used weekly step goal success in the analysis, which was defined as participants meeting 5 or more of their daily step goals each week.

### Measures

#### Real-Time Weekly Self-efficacy Assessment Via the Study Trial App

On the randomization day and at the end of each week (eg, 7 days and 14 days post randomization), between 11 AM and 3 PM, the participants were prompted the following message on their phone: “Congratulations! You made it through Week 1! How much confidence do you have that you will achieve your weekly goal this week?” The participants were able to rate their levels of confidence in real time by selecting a number from 0 to 3: “0”=not confident, “1”=a little confident, “2”=confident, and “3”=very confident. If the participants did not respond to this message before 7 PM, this question disappeared from the trial app. In this case, we coded it as a missing datum. Time stamped participant responses were transmitted via the trial app and stored in the study server.

#### Objectively Measured PA

An Active Style Pro HJA-350IT triaxial accelerometer (Omron Healthcare Co, Ltd) was used in this study [20,21]. The participants were asked to wear this device every day during the study period, except during water activities and in the shower. This accelerometer automatically reset the step counts at midnight and recorded the metabolic equivalent of tasks over 60-second epochs. Activity data from the most recent 150 days were automatically stored and directly downloaded to a computer in our research office. Details of its technical specifications and validation have been previously reported [20,21]. Before initiation of the study, we specified that the criterion for acceptable accelerometer data was at least 8 hours of wear time in a 24-hour period, 4 days per week. If a participant wore the accelerometer for at least 8 hours and 4 days a week, the adherence was calculated as 100. We then calculated the average adherence for every 6-week period. Adherence to wearing the accelerometer was on average 96.2 (SD 8.83) between baseline and 6 weeks and 96.6 (SD 12.1) between 6 weeks and 12 weeks. Average weekly step counts were calculated and compared with weekly step goals to determine weekly step goal success.

#### Other Measures

Patient weight and height were obtained by the research staff after the patients changed into a hospital gown and removed their shoes. BMI was calculated by dividing the body weight (in kg) by the square of the height (in m^2^). Sociodemographic characteristics (eg, age and education), lifestyle characteristics (eg, gym membership, diet, and weight loss plan), and self-reported cardiovascular risk factors (eg, hypertension and diabetes) were collected at the screening or baseline visit. We used the Center for Epidemiological Studies Depression Scale (CES-D), which is widely used to assess depressive symptoms in a research context [22]. It contains 20 items about symptoms that occurred in the week before the screening with response options from 0 to 3, which refer to the frequency of the symptoms. Possible total scores range from 0 to 60, with high scores indicating greater depressive symptoms. A cutoff score of 16 aids in identifying individuals at risk of clinical depression.

### Statistical Analysis

Descriptive statistics were used to describe participants' sociodemographic information, medical history, real-time weekly self-efficacy, and weekly step goal success. We used a logistic mixed model to estimate the direct effect of weekly step goal success over 12 weeks on real-time weekly self-efficacy using R (version 4.1.0) [23] with the package lme4 (version 1.1.27.1) [24]. Additional analysis using a logistic mixed model was conducted to estimate the direct effect of real-time weekly self-efficacy on weekly step goal success.

The 4-point Likert scale’s real-time weekly self-efficacy outcome was dichotomized into being confident or very confident vs being little or not confident, as only 1.6% of them reported the very lowest end of the scale (“not confident”) and no one reported the highest value of the scale (“very confident”). Additionally, potential confounders such as age, BMI, race, education, marital status, and employment status were included in the logistic mixed model. This analysis was considered valid if the data were missing at random.

Given that a total of 30.5% of real-time weekly self-efficacy measurements were missing, we were concerned that the data would be missing not at random; that is, individuals who were “not confident” to engage in PA behaviors may be more likely to avoid reporting their weekly self-efficacy. We, therefore, conducted a series of sensitivity analyses to assess how different patterns of missing data might affect the outcome of real-time weekly self-efficacy. First, we repeated the analysis setting the missing outcome to be (1) not confident, (2) confident, (3) carrying forward from previous, (4) carrying backward from subsequent, (5) setting to the mode for an individual, and (6) setting to the mode for an individual stratified by whether they met step counts. Second, we conducted multiple imputation analyses using mice (version 3.13.0) with 5 imputations [25]. Third, we fit a multinomial mixed effects logistic regression implemented through the generalized structure equation model command in Stata (version 16.1) [26], adding missing weekly self-efficacy data as an additional outcome category in the model. Additionally, we conducted sensitivity analyses to examine how different patterns of missing data affect the outcome of weekly step goal success.

## Results

### Baseline Sociodemographic and Medical Information

Table 1 shows the baseline characteristics of 140 participants receiving the 12-week intervention. The mean age was 52.7 (SD 11.5, range 25-68) years. Of the 140 participants, 83 (59.3%) were White, 28 (20.0%) were Asian or Pacific Islander, and 29 (20.7%) were African American, Hispanic or Latino, or more than one race. In addition, 112 (80.0%) had a bachelor's or advanced degree, 66 (47.1%) were married or cohabitating, and 109 (77.9%) were employed. Regarding PA-related measures, 74 (52.9%) reported previous pedometer use, 87 (62.1%) had previously participated in a diet or weight loss plan, and 41 (29.3%) had a gym membership. Lastly, the mean BMI was 29.7 (SD 6.3) kg/m^2^, 2 (1.4%) participants were smokers, and 84 (60.0%) had reached menopause. The average rating of participants' general health status was 5.1 (SD 1.0, range 1-7); 36 (25.7%) reported having high blood pressure, 48 (34.3%) had high total cholesterol, and 50 (35.7%) had CES-D scores higher than 16 points or were taking an antidepressant.

**Table 1:** Participant sociodemographics and medical history (n=140).

Characteristic			Value
Age (years), mean (SD; range)			52.7 (11.5; 25-68)
**Race or ethnicity, n (%)**			
	Asian and Pacific Islander	28 (20.0)	
	White	83 (59.3)	
	African American, Hispanic or Latino, or more than 1 race	29 (20.7)	
**Education, n (%)**			
	Completed high school or some college	28 (20.0)	
	Completed college (4 years) or graduate school	112 (80.0)	
**Annual household income (before tax; US $), n (%)**			
	<40,000	19 (13.6)	
	40,001-75,000	37 (26.4)	
	>75,000	74 (52.9)	
	Decline to state or do not know	10 (7.1)	
**Marital Status, n (%)**			
	Never married	43 (30.7)	
	Currently married or cohabitating	66 (47.1)	
	Divorced or widowed	31 (22.1)	
Employed for pay (full or part-time), n (%)			109 (77.9)
Previous pedometer use, n (%)			74 (52.9)
Drives a car at least once per week, n (%)			115 (82.1)
Participated in a diet or weight loss plan, n (%)			87 (62.1)
Has a gym membership, n (%)			41 (29.3)
**Medical history**			
	BMI (kg/m^2^)	29.7 (6.3)	
	Current smoker, n (%)	2 (1.4)	
	Reached menopause, n (%)	84 (60.0)	
	General health status, mean (SD; range)	5.1 (1.0; 3-7)	
	High blood pressure, n (%)	36 (25.7)	
	High total cholesterol, n (%)	48 (34.3)	
	CES-D^a^ score of >16 points or taking an antidepressant, n (%)	50 (35.7)	

^a^CES-D: Center for Epidemiologic Studies Depression Scale.

Table 2 presents descriptive statistics of real-time weekly self-efficacy collected by the trial app and weekly step goal success. Regarding weekly self-efficacy, the majority (71.4%) of participants reported that they were “confident/very confident” at the beginning of the intervention. However, the proportion of participants who reported “confident/very confident” at the end of week 2 decreased to 50.7%. Until the end of the intervention, the proportion of participants who reported “confident/very confident” in meeting weekly step goals remained between 38.6% and 55.0%. On the other hand, the percentage of people who indicated “not confident/a little confident” in meeting weekly step goals increased from 9.3% (beginning of the intervention) to 33.6% by week 5 and decreased to 18.6% by the end of the intervention. The pattern of real-time weekly self-efficacy showed that participants were more likely to be confident in meeting weekly goals initially and became less confident over time.

The participants’ actual performance in meeting weekly step goals shows similar patterns, as demonstrated in Table 2. At the end of week 1, overall, 64.3% of the participants met the weekly goal, meaning that they reached the step goal at least 5 days a week. In week 2, fewer participants met the weekly goal (50.7%), and there was a continued decrease in participants who met weekly goals. By the end of the intervention (week 12), only 33.6% of participants met weekly step goals (Multimedia Appendices 1 and 2 for visual presentation of weekly self-efficacy and weekly step goal outcomes).

**Table 2:** Weekly self-efficacy (confidence) and weekly step goal outcomes collected by the trial app (n=140).

		Distribution of weekly self -efficacy, n (%)	Weekly step goal outcomes, n (%)		
			Meeting weekly step goals	Not meeting weekly step goals	Missing weekly step goal outcomes
**Day 1 (randomization)**					
	Not confident or a little confident	13 (9.3)	N/A^a^	N/A	N/A
	Confident or very confident	100 (71.4)	N/A	N/A	N/A
	No response or missing	27 (19.3)	N/A	N/A	N/A
**Day 7 (week 1)**			90 (64.3)	47 (33.6)	3 (2.1)
	Not confident or a little confident	20 (14.3)	14 (70.0)	4 (20.0)	2 (10.0)
	Confident or very confident	100 (71.4)	69 (69.0)	30 (30.0)	1 (1.0)
	No response or missing	20 (14.3)	7 (35.0)	13 (65.0)	0 (0)
**Day 14 (week 2)**			71 (50.7)	66 (47.1)	3 (2.1)
	Not confident or a little confident	39 (27.9)	16 (41.0)	22 (56.4)	1 (2.6)
	Confident or very confident	71 (50.7)	45 (63.4)	26 (36.6)	0 (0)
	No response or missing	30 (21.4)	10 (33.3)	18 (60.0)	2 (6.7)
**Day 21 (week 3)**			60 (42.9)	79 (56.4)	1 (0.7)
	Not confident or a little confident	47 (33.6)	12 (25.5)	34 (72.3)	1 (2.1)
	Confident or very confident	58 (41.4)	35 (60.3)	23 (39.7)	0 (0)
	No response or missing	35 (25.0)	13 (37.1)	22 (62.9)	0 (0)
**Day 28 (week 4)**			48 (34.3)	90 (64.3)	2 (1.4)
	Not confident or a little confident	33 (23.6)	6 (18.2)	27 (81.8)	0 (0)
	Confident or very confident	77 (55.0)	34 (44.2)	43 (55.8)	0 (0)
	No response or missing	30 (21.4)	8 (26.7)	20 (66.7)	2 (6.7)
**Day 35 (week 5)**			47 (33.6)	89 (63.6)	4 (2.9)
	Not confident or a little confident	47 (33.6)	9 (19.1)	37 (78.7)	1 (2.1)
	Confident or very confident	56 (40.0)	30 (53.6)	26 (46.4)	0 (0)
	No response or missing	37 (26.4)	8 (21.6)	26 (70.3)	3 (8.1)
**Day 42 (week 6)**			39 (27.9)	98 (70.0)	3 (2.1)
	Not confident or a little confident	32 (22.9)	9 (28.1)	23 (71.9)	0 (0)
	Confident or very confident	54 (38.6)	22 (40.7)	32 (59.3)	0 (0)
	No response or missing	54 (38.6)	8 (14.8)	43 (79.6)	3 (5.6)
**Day 49 (week 7)**			52 (37.1)	85 (60.7)	3 (2.1)
	Not confident or a little confident	31 (22.1)	7 (22.6)	24 (77.4)	0 (0)
	Confident or very confident	73 (52.1)	38 (52.1)	34 (46.6)	1 (1.4)
	No response or missing	36 (25.7)	7 (19.4)	27 (75.0)	2 (5.6)
**Day 56 (week 8)**			49 (35.0)	86 (61.4)	5 (3.6)
	Not confident or a little confident	24 (17.1)	5 (20.8)	17 (70.8)	22 (91.7)
	Confident or very confident	64 (45.7)	34 (53.1)	30 (46.9)	0 (0)
	No response or missing	52 (37.1)	10 (19.2)	39 (75.0)	3 (5.8)
**Day 63 (week 9)**			47 (33.6)	86 (61.4)	7 (5.0)
	Not confident or a little confident	25 (17.9)	4 (16.0)	20 (80.0)	1 (4.0)
	Confident or very confident	65 (46.4)	33 (50.8)	31 (47.7)	1 (1.5)
	No response or missing	50 (35.7)	10 (20.0)	35 (70.0)	5 (10.0)
**Day 70 (week 10)**			43 (30.7)	91 (65.0)	6 (4.3)
	Not confident or a little confident	22 (15.7)	3 (13.6)	18 (81.8)	1 (4.5)
	Confident or very confident	57 (40.7)	28 (49.1)	29 (50.9)	0 (0)
	No response or missing	61 (43.6)	12 (19.7)	44 (72.1)	5 (8.2)
**Day 77 (week 11)**			42 (30.0)	90 (64.3)	8 (5.7)
	Not confident or a little confident	23 (16.4)	1 (4.3)	22 (95.7)	0 (0)
	Confident or very confident	58 (41.4)	25 (43.1)	31 (53.4)	2 (3.4)
	No response or missing	59 (42.1)	16 (27.1)	37 (62.7)	6 (10.2)
**Day 83 (week 12)**			47 (33.6)	85 (60.7)	8 (5.7)
	Not confident or a little confident	26 (18.6)	7 (26.9)	18 (69.2)	1 (3.8)
	Confident or very confident	65 (46.4)	31 (47.7)	34 (52.3)	0 (0)
	No response or missing	49 (35.0)	9 (18.4)	33 (67.3)	7 (14.3)

^a^N/A: not applicable.

### Association Between Real-time Weekly Self-efficacy and Weekly PA Outcomes

Table 3 presents the results of a multivariable logistic mixed model predicting the longitudinal outcome of real-time weekly self-efficacy. The participants who met their weekly step goals were 4.41 times more likely to be self-efficacious about meeting the following week's step goals than those who did not (adjusted odds ratio [OR] 4.41; 95% CI 2.59-7.50; *P*<.001) even after controlling for age, BMI, race, education, marital status, and employment status. Moreover, additional analysis revealed that participants who were confident or very confident about meeting the following week’s step goals were 2.07 times more likely to meet their weekly step goals in the following week (OR 2.07, 95% CI 1.16-3.70; *P*=.01).

**Table 3:** Multivariable logistic mixed models of the longitudinal outcome of real-time weekly self-efficacy and weekly step goal success.

Predictors	Outcome: weekly self-efficacy		Outcome: weekly step goal success	
	Adjusted odds ratio (95% CI)	*P* value	Adjusted odds ratio (95% CI)	*P* value
Weekly step goal success^a^	4.41 (2.59-7.50)	<.001	—^b^	—
Weekly self-efficacy	—	—	2.07 (1.16-3.70)	.01
Age	1.04 (1.00-1.08)	.03	1.13 (1.07-1.19)	<.001
BMI	1.02 (0.96-1.09)	.56	1.10 (0.94-1.10)	.73
Non-White^c^	Reference	N/A^d^	Reference	N/A
White	0.48 (0.21-1.12)	.09	0.41 (0.13-1.28)	.12
High school or some college	Reference	N/A	Reference	N/A
College or graduate degree	1.35 (0.48-3.78)	.57	0.35 (0.09-1.30)	.12
Never married, divorced, or widowed	Reference	N/A	Reference	N/A
Married or cohabitated	0.86 (0.40-1.83)	.69	1.34 (0.50-3.60)	.56
Unemployed	Reference	N/A	Reference	N/A
Employed full- or part-time	0.56 (0.22-1.46)	.24	0.82 (0.24-2.82)	.75

^a^Weekly step goal success was measured across 13 weeks.

^b^Not determined.

^c^Non-White included Asian, Pacific Islander, African American, Hispanic or Latino, and multiracial individuals.

^d^N/A: not applicable.

Since 14.3%-43.6% of the participants did not respond to the real-time weekly self-efficacy question (Table 2), we conducted a series of sensitivity analyses (described in the *Statistical Analysis* section) to assess the impact of missing data. As shown in Table 4, the sensitivity analyses still supported a strong effect of weekly step goal success on real-time weekly self-efficacy outcomes, with adjusted OR estimates ranging from 2.31 (95% CI 1.50-3.56) to 4.66 (95% CI 2.72-7.99). Additional sensitivity analyses found similar patterns regarding the effect of real-time weekly self-efficacy on weekly step goal success with OR estimates ranging from 1.44 (95% CI 0.87-2.40) to 2.22 (95% CI 1.39-3.80).

**Table 4:** Sensitivity analyses of predicting real-time weekly self-efficacy and weekly step goal success.

Model type^a^			Different definition of missing self-efficacy Outcome condition		Outcome: weekly self-efficacy; predictor: weekly step goal success				Outcome: weekly step goal success; predictor: weekly self-efficacy				
					Adjusted odds ratio (95% CI)		*P* value		Adjusted odds ratio (95% CI)			*P* value	
**Logistic mixed model^b^**													
	Model 1	Missing cases of self-efficacy were set to “not confident”		3.55 (2.55-4.96)		<.001		1.69 (1.12-2.54)			.01		
	Model 2	Missing cases of self-efficacy were set to “confident”		2.31 (1.50-3.56)		<.001		1.44 (0.87-2.40)			.16		
	Model 3	Missing cases of self-efficacy were set to the value from previously reported self-efficacy		4.40 (2.60-7.46)		<.001		1.98 (1.17-3.36)			.01		
	Model 4	Missing cases of self-efficacy were set to the value from subsequently reported self-efficacy		3.90 (2.34-6.50)		<.001		2.13 (1.25-3.63)			.005		
	Model 5	Missing cases of self-efficacy were set to the most common self-efficacy value of the individual		3.30 (1.98-5.52)		<.001		2.12 (1.24-3.64)			.006		
	Model 6	Missing cases of self-efficacy were set to the most common self-efficacy value of the individual stratified by whether they met weekly step counts		4.66 (2.72-7.99)		<.001		2.22 (1.39-3.80)			.004		
	Model 7	Missing cases of self-efficacy were set using multiple imputation		4.40 (2.59-7.50)		<.001		2.07 (1.16-1.96)			.01		
**Multinomial mixed model^b^**										1.76 (1.02-3.01)			.04
	Model 8	Missing cases of self-efficacy were set as a separate outcome category		2.79 (1.85-4.22)		<.001		1.69 (1.12-2.54)			.01		

^a^All models are also adjusted for age, BMI, self-reported White race, college education or higher, being married, and employment status.

^b^The models presented are independent of one another.

## Discussion

### Principal Findings

This study examined a novel approach for the assessment of the direct association between individuals’ weekly step goal success and real-time self-efficacy using our cellphone app. One notable finding of the study was that individuals who met weekly step goals were more likely to be self-efficacious about meeting weekly step goals for the upcoming week, even after controlling for potential confounding factors. A potential explanation for this finding is related to the sources of self-efficacy. According to Bandura [7,17], one’s self-efficacy develops through 4 primary sources of influence. These include mastery experiences (eg, personal success), vicarious experiences provided by social models (eg, seeing people similar to oneself succeed), social persuasion (eg, verbal positive appraisals of capabilities), and somatic and emotional states (eg, positive mood) [17]. Of these, mastery experience is considered the greatest source of one's self-efficacy. Thus, participants who successfully met weekly step goals may have established a strong sense of self-assurance that they will be able to meet the following week's step goals. Additional analysis revealed that real-time weekly self-efficacy was a significant predictor of weekly step goal success, suggesting a bidirectional association between self-efficacy and step goal outcomes. Interestingly, findings show that the effect of weekly step goal success on weekly self-efficacy was larger than the effect of weekly self-efficacy on weekly step goal success. Given that the effect of self-efficacy on PA outcomes was mainly highlighted in previous empirical studies [27,28], our study suggests that future research may benefit from examining the direct effects of PA outcomes on one’s self-efficacy.

Self-efficacy measured at a limited set of time points (eg, baseline and 12 weeks) is frequently used to predict PA in intervention studies [29]. However, the findings of a systematic review were inconclusive in showing an association between self-efficacy and PA [30]. This finding may be attributed to the lack of a standardized definition of self-efficacy across studies. For example, studies used different self-efficacy definitions, such as one’s confidence in one’s ability to engage in specific activities (eg, walking), and self-efficacy in overcoming barriers to PA [31-33]. In our study, we show that tracking real-time confidence in the participants’ ability to meet the upcoming weekly step goal is an excellent predictor of PA. Additionally, our results demonstrate that participants’ confidence fluctuates over time, particularly showing a decline in the first 3 weeks of the intervention. Similar results have been reported in previous literature [29]. In our study, given that the step goals automatically increased by 20% each week regardless of participants’ progress, their lack of control over the weekly step goals may have contributed to a decline in their confidence. Thus, to prevent the decline in self-efficacy, tailored PA goals based on participants’ progress or preferences are recommended for future PA interventions. In addition, our results suggest the potential to intervene in PA relapse in real time in individuals who are struggling to maintain regular PA, although future interventional studies are warranted to further confirm these findings.

In this study, we performed real-time data collection via a mobile app over a 12-week period to address the limitations of traditional self-efficacy assessments and to offer novel insights into the determinants of PA behavior. According to Dunton [8], the EMA approach may advance the understanding of PA behavior in 3 criterial ways: synchronicity, sequentiality, and instability. Weekly assessment of momentary information regarding participants' perceptions of their self-efficacy and objectively measured PA outcomes help understand intraindividual (ie, within-person) effects that operate across time (ie, week to week). In contrast, a traditional intermittent assessment may limit the ability to detect temporal and spatial variations. Sequentiality provides information concerning the temporal sequence between weekly step goal success and perceptions of self-efficacy toward meeting the following week's step goals. For instability, EMA allows us to capture the dynamic and fluctuating patterns of weekly self-efficacy and step goal success. For example, 2 study participants may have the same self-efficacy level at baseline and post intervention in a traditional assessment method. However, the extent of fluctuation and variation between the 2 time points may differ. We believe that this study’s findings shed light on this information that is often missing through the use of our novel approach using EMA.

### Strengths and Limitations

The strengths of this study include the accuracy of accelerometer-measured daily activity, excellent adherence to wearing an accelerometer, and low dropout rate during the 12-week intervention. However, several limitations should be acknowledged. First, our linear mixed model for self-efficacy assumed that the data were missing at random. One may argue that the outcome might not be missing at random and there is, in fact, a systemic bias (ie, those with missing outcomes are more likely to have low self-efficacy). To address this limitation, we conducted extensive sensitivity analyses to mitigate this possibility, all indicating a strong effect. Second, only female adults motivated to participate in a lifestyle modification program were enrolled in the study. Thus, the results may not be generalizable to male adults or children. Lastly, self-efficacy measured in this study was specific to meeting weekly step goals. Therefore, it may not reflect one’s global self-efficacy for being physically active. Future research may benefit from comparing different types of self-efficacy measures to predict PA outcomes.

### Conclusions

This study demonstrates the potential utility of a novel approach to examine self-efficacy in real time for analysis of self-efficacy in conjunction with objectively measured PA. Discovering the dynamic patterns and changes in weekly self-efficacy and PA behaviors within participants and between participants may aid in designing a more personalized PA intervention than programs that are currently available. Evaluation of this novel approach in an RCT is warranted.

## Appendix

Multimedia Appendix 1 Changes in self-efficacy (confident/very confident) and step goal outcomes (meeting step goals) across 12 weeks (N = 140).

Multimedia Appendix 2 Changes in self-efficacy and step goal outcomes across 12 weeks (N = 140).

## Abbreviations

CES-D: Center for Epidemiological Studies Depression Scale
EMA: Ecological Momentary Assessment
OR: odds ratio
PA: physical activity
RCT: randomized controlled trial
