# Supplementary material for: A Novel Approach to Assess Weekly Self-efficacy for Meeting Personalized Physical Activity Goals Via a Cellphone: 12-Week Longitudinal Study
Source: JMIR Form Res. 2023 Jan 27;7:e38877. doi: 10.2196/38877 (PMC9919464; doi:10.2196/38877)
Supplement: Multimedia Appendix 1 [file formative_v7i1e38877_app1.docx]

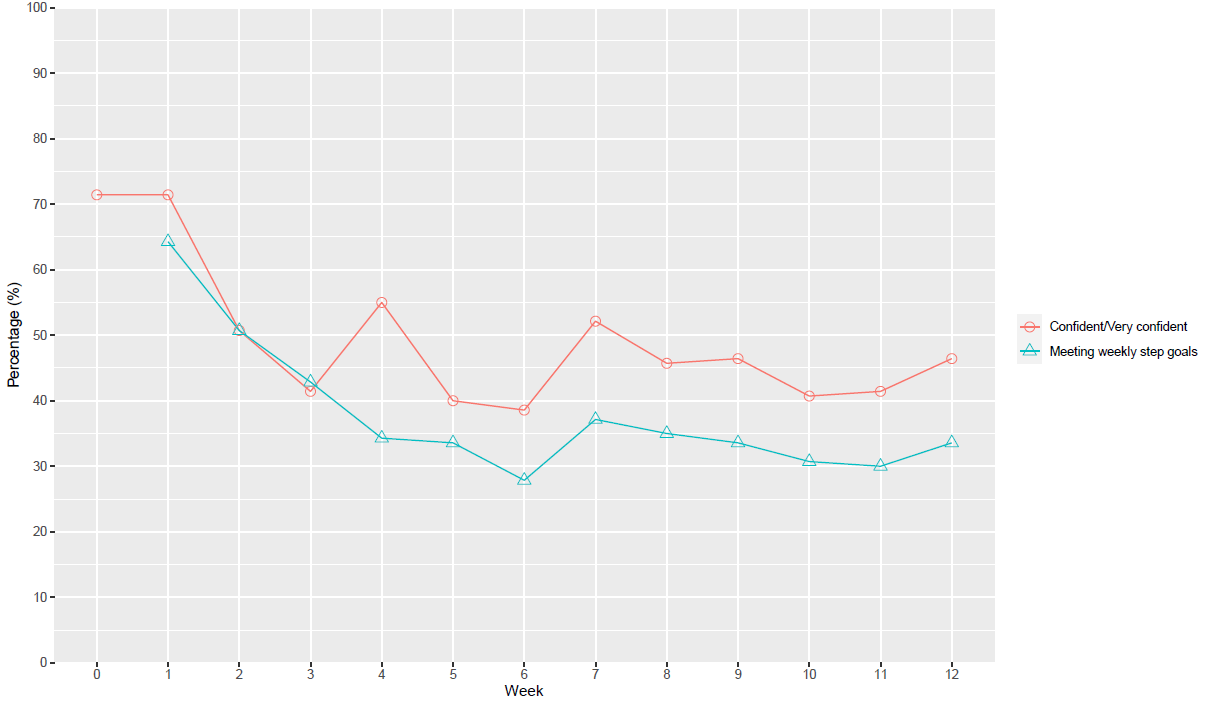


Changes in self-efficacy (confident/very confident) and step goal outcomes (meeting step goals) across 12 weeks (N = 140).
