# Supplementary figures and images for: A Novel Approach to Assess Weekly Self-efficacy for Meeting Personalized Physical Activity Goals Via a Cellphone: 12-Week Longitudinal Study
Source: JMIR Form Res. 2023 Jan 27;7:e38877. doi: 10.2196/38877 (PMC9919464; doi:10.2196/38877)

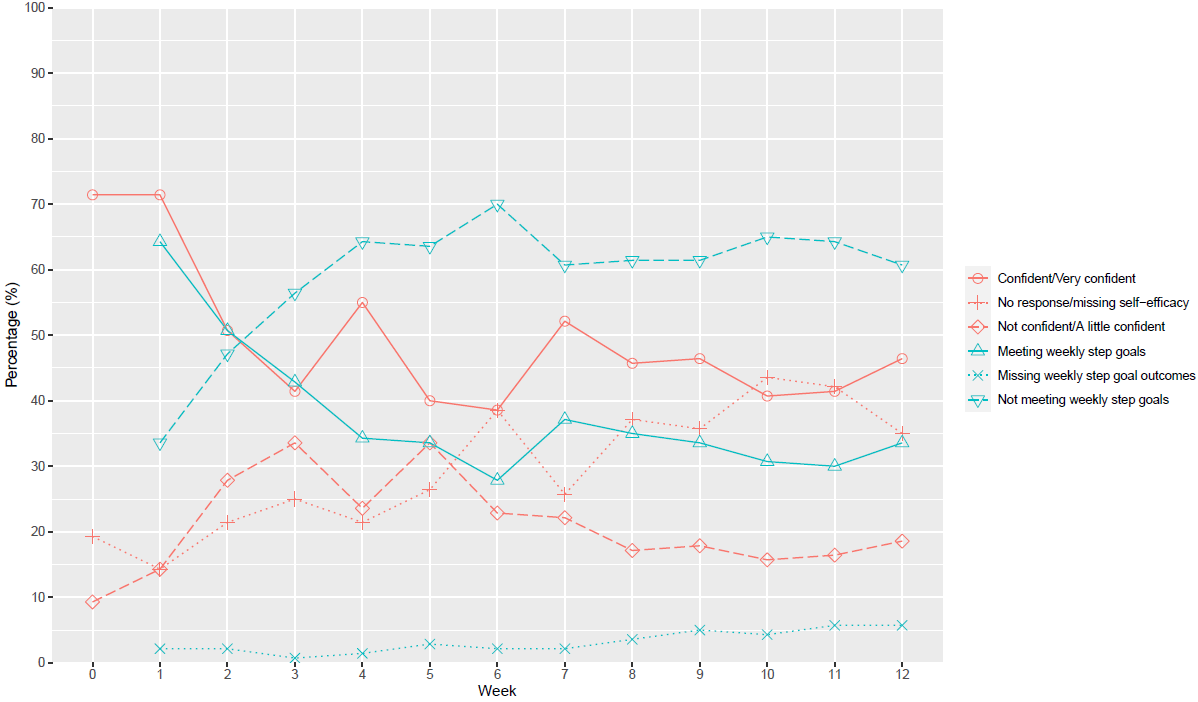


Changes in self-efficacy and step goal outcomes across 12 weeks (N = 140).

Supplement: Multimedia Appendix 2 [file formative_v7i1e38877_app2.docx]
